# Aging exacerbates cardiac dysfunction and mortality in sepsis through enhancing TLR2 activity

**DOI:** 10.3389/fcvm.2023.1293866

**Published:** 2023-11-29

**Authors:** Yufeng Zhai, Qingzhou Yao, Erlinda The, Lihua Ao, David A. Fullerton, Xianzhong Meng

**Affiliations:** Department of Surgery, University of Colorado Denver, Aurora, CO, United States

**Keywords:** aging, sepsis, cardiac dysfunction, mortality, myocardial TLR2

## Abstract

**Introduction:**

Sepsis is prevalent in the elderly population with increased incidence and mortality. Currently, the mechanism by which aging increases the susceptibility to sepsis and worsens outcome is unclear. We tested the **hypothesis** that aging exacerbates cardiac dysfunction in sepsis through a Toll-like receptor 2 (TLR2)-dependent mechanism.

**Methods:**

Male young adult (4–6 months) and old (18–20 months) wild type (WT) and TLR2 knockout (KO) mice were subject to moderate sepsis by cecal ligation and puncture. Additional groups of young adult and old WT mice were treated with TLR2 agonist Pam3CSK4. Left ventricle (LV) performance was evaluated with a pressure-volume microcatheter. Tumor necrosis factor-α (TNF-α), interleukin (IL)-1β, IL-6 and monocyte chemoattractant protein-1 (MCP-1) in the myocardium and plasma were assessed using enzyme-linked immunosorbent assay.

**Results:**

Sepsis reduced LV ejection fraction and cardiac output in both young adult and old WT mice. However, identical CLP caused more severe cardiac dysfunction and high mortality in old WT mice that were accompanied by greater levels of TNF-α, IL-1β, IL-6 and MCP-1 in the myocardium and plasma. TLR2 KO diminished aging-related difference in myocardial and systemic inflammatory response, resulting in improved cardiac function and decreased mortality in old septic mice. In addition, higher myocardial TLR2 levels in old WT mice resulted in greater myocardial inflammatory response and worse cardiac dysfunction following administration of TLR2 agonist.

**Conclusion:**

Moderate sepsis results in greater cardiac dysfunction and significant mortality in old mice. Aging elevates TLR2 level/activity to exacerbate the inflammatory response to sepsis, leading to worse cardiac dysfunction and mortality.

## Introduction

Sepsis is a condition where infection serves as an initial trigger for the systemic inflammatory response syndrome (SIRS), and can cause multiple organ dysfunction, shock and death (1). The incidence of sepsis is greater in the elderly population, and elderly patients with sepsis often have more severe organ dysfunction and higher mortality rate (2, 3). For instance, septic patients aged 65 or older face a mortality rate of 30%–40%, whereas younger patients have a mortality rate of 4%–5% (4). The higher mortality in older septic patients is closely associated with organ dysfunction, but the underlying mechanism responsible for aging-related susceptibility to organ impairment and severity of organ dysfunction remains incompletely understood.

Sepsis is defined by an inflammatory response driven by the host's immune system reacting to an infection. The innate immune response in septic patients is highly dynamic and elicited by various factors, including pathogen-associated molecular patterns (PAMPs) like bacterial antigens, and damage-associated molecular patterns (DAMPs), which are proteins released from injured cells (5). Systemic activation of the innate immune system by PAMPs and DAMPs leads to elevated and persistent inflammatory responses that are characterized by exaggerated production and release of inflammatory mediators, such as TNF-α, IL-1β, IL-6 and MCP-1 (6, 7). Cardiac dysfunction is a common manifestation of organ impairment in sepsis and plays a crucial role in the complex pathophysiology of septic shock, contributing to tissue perfusion reduction, multiple organ failure and death (8). Previous studies indicates that exacerbated production of inflammatory mediators, including IL-6 and MCP-1 in the myocardium, plays a mechanistic role in cardiac dysfunction induced by endotoxemia or sepsis (9, 10). Furthermore, certain inflammatory mediators, such as TNF-α and IL-1β, synergistically depress cardiac contractility (11). Thus, dysregulated inflammatory responses occupy an important role in mediating cardiac dysfunction. However, the impact of aging on the myocardial inflammatory responses to sepsis has not been defined.

Toll-like receptors (TLRs) are highly conserved pattern recognition receptors responsible for initiating innate immune responses against various pathogens. These receptors play a critical role in recognition of exogenous and endogenous danger signals, including PAMPs and DAMPs. TLR2, in particular, can recognize both bacterial PAMPs and endogenous DAMPs, such as heat shock proteins and high mobility group box 1, which are released as a result of tissue damage and necrosis. Upon binding with ligand, activated TLR2 initiates an intracellular signaling cascade that leads to the activation of MAPK and NF-κB, and results in the production of inflammatory chemokines and cytokines, reactive oxygen species and acute-phase proteins (5). Although TLR signaling is essential for host defense, persistent activation of TLRs, particularly TLR2, and related signaling pathways during sepsis would result in dysregulated systemic inflammatory responses (12). We observed in a previous study that aging elevates myocardial TLR2 levels (13), indicating that elevated TLR2 activity in aging heart augments myocardial inflammatory responses to sepsis. The current study tested the hypothesis that TLR2 plays a critical role in mediating myocardial inflammatory responses and cardiac dysfunction in old septic mice. The aims of the present study were to determine: (1) the impact of aging on the myocardial inflammatory responses to sepsis in a cecal ligation and puncture model using old mice; (2) the effect of TLR2 knockout on the myocardial inflammatory responses in old septic mice; (3) the contribution of TLR2-mediated myocardial inflammatory responses to cardiac dysfunction and mortality caused by sepsis in old mice.

## Materials and methods

### Chemicals and reagents

Antibodies against TLR2 and β-actin were procured from Santa Cruz Biotechnology (Dallas, TX). Enzyme-linked immunosorbent assay (ELISA) kits for MCP-1, IL-6, TNF-α and IL-1β were procured from R&D System (Minneapolis, MN). Pam3CSK4 was procured from InvivoGen (San Diego, CA). All other chemicals and reagents were procured from Sigma-Aldrich Chemical (St Louis, MO).

### Animals

Male old (18–20 months) C57BL/6 mice were sourced from the National Institute on Aging (Bethesda, MD, USA). Male young adult (4–6 months) C57BL/6 mice and male TLR2 KO mice (B6.129-Tlr2^tm1Kir^/J) were procured from Jackson Laboratory (Bar Harbor, Maine, USA). TLR2 KO mice were housed in the Animal Care Center until to be 18–20-month-old. The TLR2 knockout mice are homozygous, develop normally and exhibit no physiological abnormalities. Mice were assigned into experimental groups and underwent to cecal ligation and puncture (CLP), sham procedure. For TLR2 activity experiment, young adult and old C57BL/6 mice were treated with TLR2 agonist Pam3CSK4 (2.5 µg/ g, iv).

### Cecal ligation and puncture

Mice were anesthetized with 3%–5% isoflurane for induction and maintained with 1.5%–2% isoflurane using a nose cone. After buprenorphine SR was administered subcutaneously, the abdomen is shaved and disinfected with betadine and alcohol. A small longitudinal skin incision, approximately 1.0 cm left from the midline, was made. The cecum was moved out of peritoneal cavity, 33% of the cecum was ligated with 7-0 surgical sutures. The ligated cecum was punched once with a 27-gauge needle to create a moderate sepsis. After the puncture, a small of quantity of fecal material was deliberately expelled from the puncture site to ensure its openness. Sham animals were subjected to laparotomy with cecum extrusion, but without ligation and puncture. The cecum was placed back to the abdomen after manipulation, and the incisions in the peritoneal wall and skin were sutured shut. Normal saline (1.0 ml) was injected subcutaneously to keep the animal from dehydration. Then the animals were returned to their cages for recovery. Accumulated mortality over 4 days after CLP was determined.

### Pressure-volume hemodynamic analysis

The left ventricle (LV) function was analyzed in survivors 1, 2 and 4 days after CLP. The measurement was conducted following the protocol described previously (14). Briefly, mice were anesthetized with isoflurane inhalation (2%), and anticoagulated with heparin (Elkins-Sinn, Cherry Hill, NJ; 1,000 units/kg, ip). Animals were positioned in a supine manner on a warming pad, ensuring a core body temperature was upheld at 37 ± 0.5°C. Subsequently, a pressure-volume microcatheter (ADInstruments Ltd, Colorado Springs, CO) was introduced into the left ventricle through the right common carotid artery. Pressure-volume loops were recorded for 20 min with the MPVS-400 System and PVAN software (Millar Instruments, Houston, TX). Heart rate, LV developed pressure, ejection fraction, and cardiac output were determined. Animals were sacrificed after measurement of cardiac function. The heart tissue were prepared for further analysis.

### Cytokine assay

ELISA kits were employed to quantify MCP-1, IL-6, TNF-α, and IL-1β in blood and myocardial tissue. Samples and standards were readied in accordance with the manufacturer's guidelines. Absorbance of both standards and samples was measured spectrophotometrically at 450 nm utilizing a microplate reader (Bio-Rad Laboratories, Inc, Hercules, CA).

### Immunoblotting

TLR2 levels in the heart, liver and kidney were analyzed using immunoblotting. The tissue homogenate was separated on acrylamide gels with a 4%–20% gradient and subsequently transferred onto a nitrocellulose membrane (Bio-rad Laboratories, Hercules, CA). After incubation with primary and secondary antibodies, the membrane was treated with enhanced chemiluminescence reagents (Bio-rad Laboratories, Hercules, CA) to identify the protein band. The protein band image was analyzed and recorded using the ChemiDoc Imaging System (Bio-rad Laboratories, Hercules, CA). The area and intensity of protein bands were assessed using NIH Image J software (Wayne Rasband, National Institutes of Health, Bethesda, MD). These measurements were standardized against the β-actin band, which served as loading control.

### Statistical analysis

Statistical analysis was conducted utilizing StatView software from Abacus Concepts in Calabasas, CA. The data were presented as mean ± standard error (SEM). Pairwise comparisons between two groups were evaluated using the student's *t*-test, and the distinctions were further validated through the Nonparametric Mann–Whitney U test. When comparing multiple groups, ANOVA with *post hoc* Bonferroni/Dunn testing was applied, with confirmation of differences via the nonparametric Kruskal–Wallis test. A significance level of *P* < 0.05 was deemed as indicative of statistical significance.

## Results

### Old mice have severe cardiac dysfunction and high mortality during sepsis

**Figure 1:**
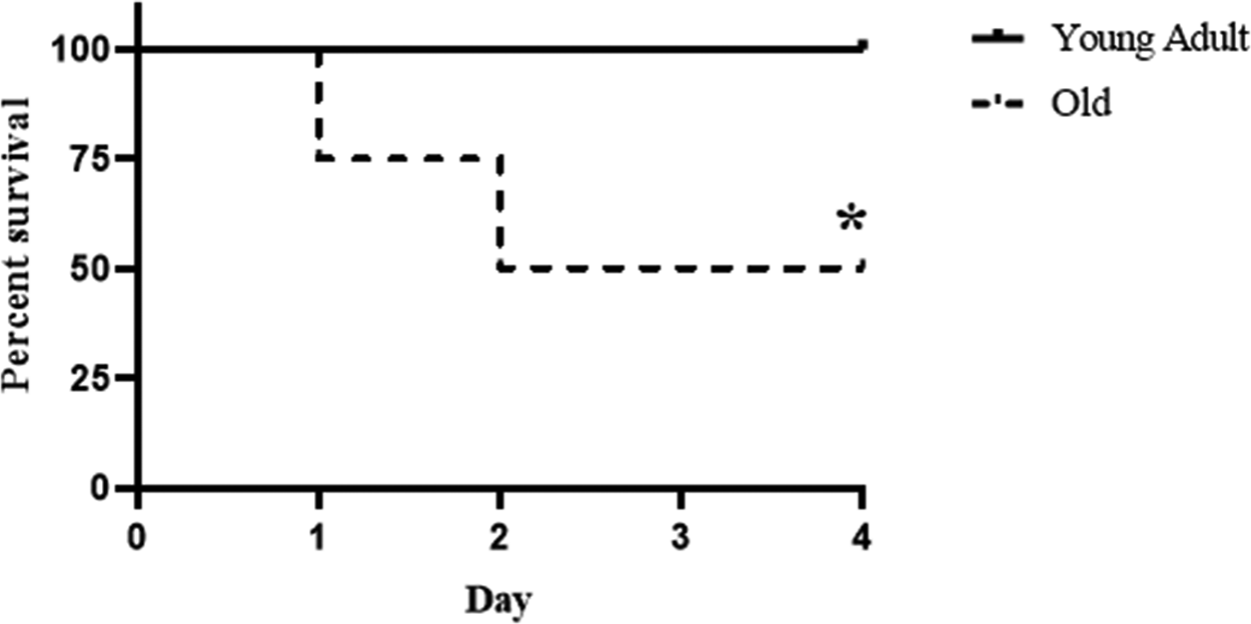
Old septic mice have high mortality. Male young adult WT mice (4–6 month old) and old WT mice (18–20 month old) were subjected to cecal ligation and puncture (CLP; 1 puncture with a 27G needle)**.** Half of the old WT mice died during the 4-day period following CLP while none of the young adult mice died in the same period. Old mice mainly die in the first 2 days. *n* = 12 in each group; *P < 0.05 *vs.* WT young adult mice.

We recorded mortality in the 4-day period following CLP. As shown in Figure 1, all young adult mice survived the moderate sepsis. However, 50% of old mice died in the first 2 days after CLP. Thus, non-lethal sepsis in young adult mice caused significant mortality in old mice.

**Figure 2:**
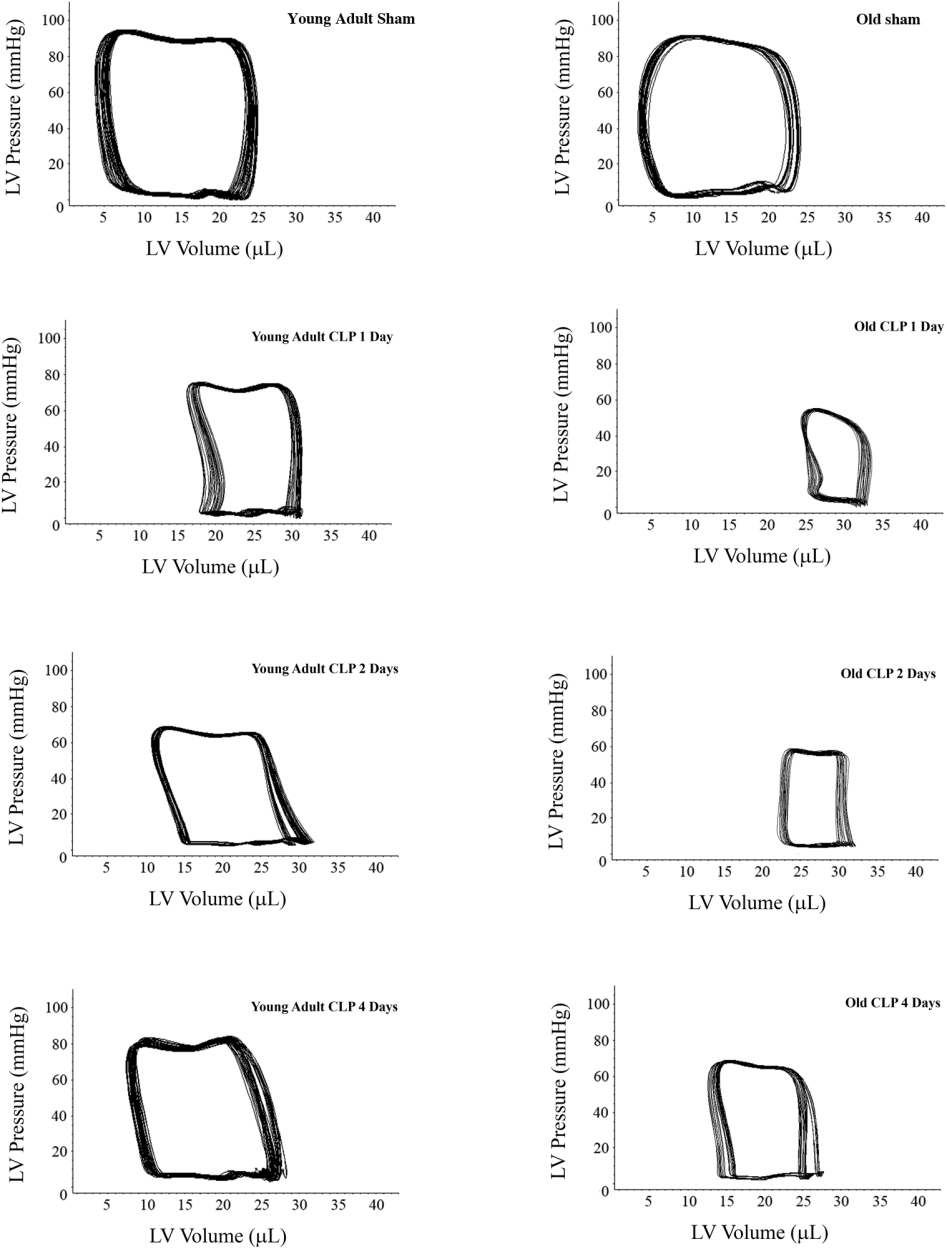
Sepsis results in greater and sustained cardiac dysfunction in old mice. Young adult and old mice were subject to CLP or sham procedure. Left ventricular (LV) function was analyzed using microcatheter at 1, 2, and 4 days following CLP. Representative pressure-volume loops show that sepsis resulted in more severe cardiac dysfunction in old mice compared to young adult mice. While cardiac function in young adult mice recovered to a level comparable to the sham control level 4 days after CLP, it remained notably lower than the corresponding sham control level in old mice after 4 days.

To evaluate the impact of aging on cardiac dysfunction in sepsis, we analyzed LV performance in survivors 1, 2 and 4 days after CLP using a microcatheter system. The pressure-volume loops presented in Figure 2 show a slight shift to the right in both young adult and old mice due to increases in end-systolic and end-diastolic volume caused by sepsis. At each time point after CLP, old mice had a significantly smaller loop area than young adult mice, indicating lower developed pressure and reduced stroke volume in old septic mice. Detailed LV function parameters are summarized in Table 1. Both young and old WT mice had decreased ejection fraction, developed pressure, and cardiac output following CLP. The lowest LV function was observed 1 day after CLP in both young adult and old mice (Table 1). Compared to age-matched sham controls, ejection fraction was approximately 70% lower (18 ± 2.0% vs. 61 ± 5%; *P* < 0.05), cardiac output 55% lower (2.4 ± 0.3 vs. 5.4 ± 0.5 ml/min; *P* < 0.05), and developed pressure 40% lower (50 ± 5 vs. 82 ± 5 mmHg; *P* < 0.05) at this time point in old mice. However, ejection fraction in young adult mice was 37% lower than age-matched sham controls (40 ± 3 vs. 64 ± 6%; *P* < 0.05), cardiac output 26% lower (4.7 ± 0.3 vs. 6.5 ± 0.5 ml/min; *P* < 0.05), and development pressure 18% lower (70 ± 8 vs. 86 ± 6 mmHg; *P* < 0.05) at the same time point in young adult mice. LV function was recovering thereafter. While LV function was fully recovered in young adult mice 4 days after CLP, it remained significantly lower in old mice at this time point (Table 1).

**Table 1:** Aging exacerbates cardiac dysfunction caused by sepsis.

	Young adult sham	Young adult CLP 1 day	Young adult CLP 2 days	Young adult CLP 4 days	Old sham	Old CLP 1 day	Old CLP 2 days	Old CLP 4 days
Heart rates (bpm)	464 ± 40	470 ± 30	432 ± 40	451 ± 42	435 ± 41	420 ± 42	410 ± 52	430 ± 21
Developed pressure (mmHg)	86.3 ± 6.1	70.5 ± 8.3^a^	74.2 ± 3.2^a^	85.7 ± 7.5	82.4 ± 5.1	50.5 ± 5.2^a^^b^	54.3 ± 4.3^a^^b^	67.4 ± 4.4^a^^b^
End-systolic volume (ul)	7.7 ± 1.5	20.1 ± 2.0^a^	16.0 ± 2.0^a^	9.0 ± 0.8	8.1 ± 0.7	27.3 ± 3.2^a^^b^	24.0 ± 2.1^a^^b^	16.0 ± 1.3^a^^b^
End-diastolic volume (ul)	21.8 ± 2.0	30.2 ± 2.5^a^	28.0 ± 2.3^a^	23.0 ± 2.5	20.0 ± 1.7	33.4 ± 3.1^a^	30.1 ± 2.5^a^	24.0 ± 2.0^a^
Ejection fraction (%)	63.8 ± 6.3	40.1 ± 3.2^a^	43.5 ± 4.1^a^	61.3 ± 5.2	61.7 ± 5.3	18.2 ± 2.1^a^^b^	20.6 ± 3.2^a^^b^	33.4 ± 2.3^a^^b^
Cardiac output (ml/min)	6.5 ± 0.5	4.7 ± 0.3^a^	5.2 ± 0.3^a^	6.3 ± 0.4	5.4 ± 0.5	2.4 ± 0.3^a^^b^	2.5 ± 0.1^a^^b^	3.4 ± 0.2^a^^b^

Young adult and old mice were subjected to CLP or sham procedure. Cardiac function was analyzed 1–4 days following CLP. Data are expressed as mean ± SE.

*n* = 6 in each group.

^a^ *P *< 0.05 vs. respective sham.

^b^ *P *< 0.05 vs. young adult at the same time point.

### TLR2 KO results in greater reduction of inflammatory activity in old septic mice

**Figure 3:**
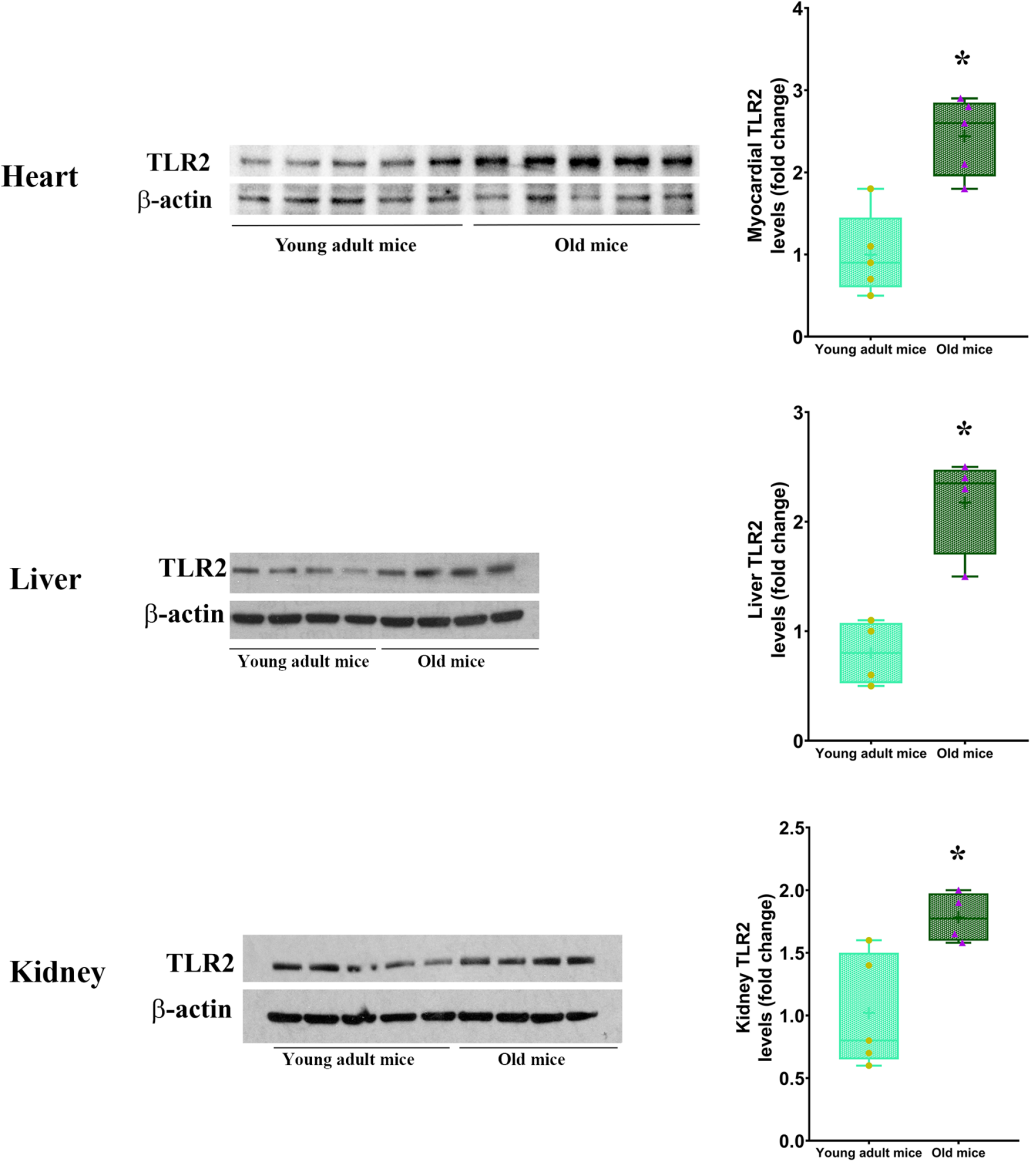
TLR2 level is elevated in multiple organs of old mice. Immunoblotting and densitometric analysis revealed higher levels of TLR2 in the heart, liver and kidney of old mice in comparison to young adult mice. Data are presented as box-and-whiskers plots where the upper and lower borders of the box represent the upper and lower quartiles; the horizontal line inside the box represents the median; the upper and lower whiskers represent the maximum and minimum values of nonoutliers; and the + sign represents the mean, *n* = 4–5; *P < 0.05 vs. young adult.

**Figure 4:**
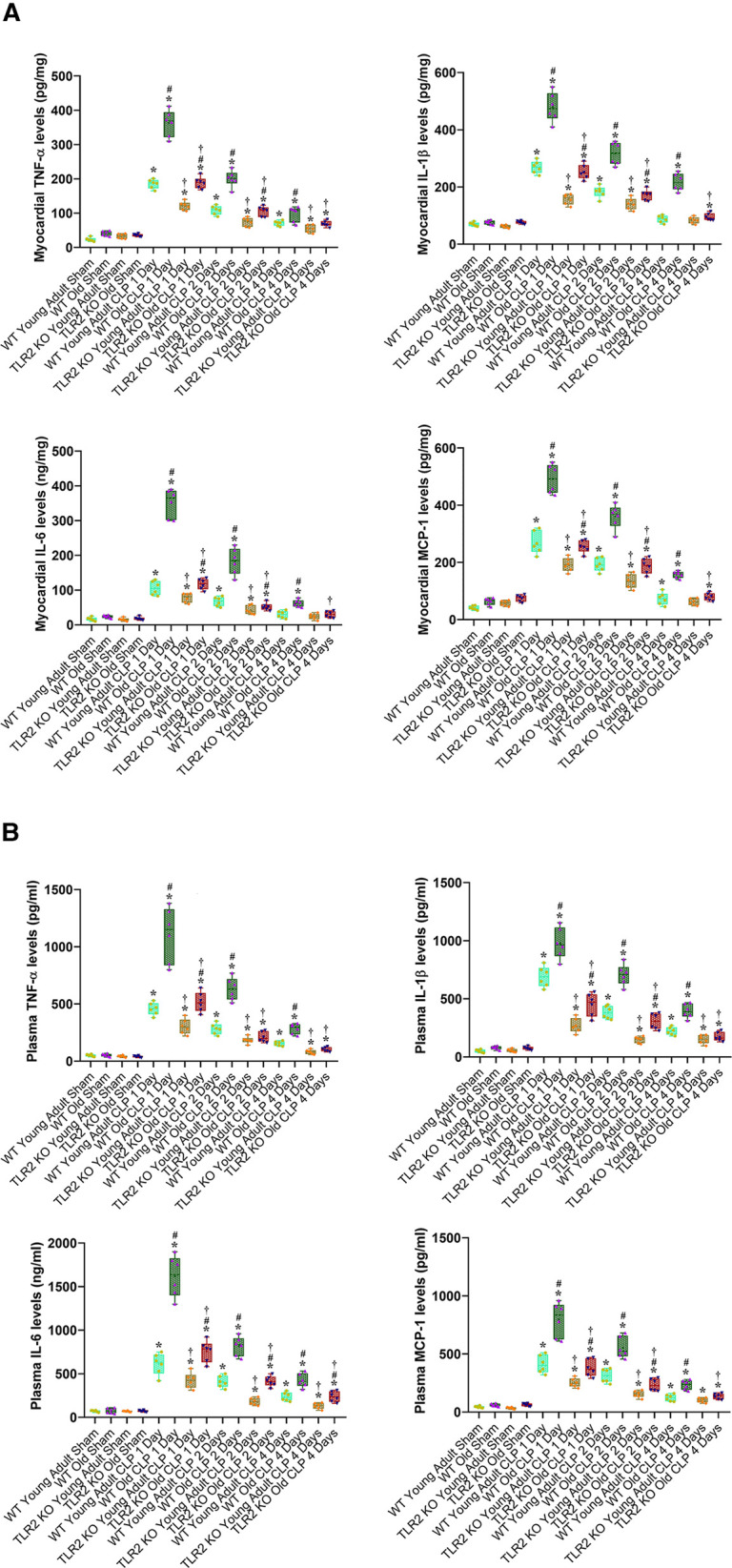
TLR2 KO results in a greater reduction of the inflammatory activity during sepsis in old mice. WT young adult and old mice, as well as TLR2 KO young adult and old mice were subjected to CLP or sham procedure. TNF-α, IL-1β, IL-6 and MCP-1 in the myocardium (**A**) and plasma (**B**) were analyzed using ELISA. Old WT mice had a greater increase in the levels of inflammatory cytokines at all of the time points following CLP. TLR2 KO attenuated the inflammatory response to sepsis in both young adult and old septic mice and markedly diminished the age-related difference in cytokine levels following CLP. Data are presented as box-and-whiskers plots where the upper and lower borders of the box represent the upper and lower quartiles; the horizontal line inside the box represents the median; the upper and lower whiskers represent the maximum and minimum values of nonoutliers; and the + sign represents the mean, *P < 0.05 vs. corresponding sham; ^#^*P *< 0.05 vs. corresponding young adult of the same strain; ^†^*P *< 0.05 vs. WT of the same age. *n* = 6 in each group.

Our previous study found that the myocardium of old mice has higher level of TLR2 and displays enhanced inflammatory response to ischemia (13). The present study found that old mice had higher TLR2 levels in the heart, liver and kidney (Figure 3). To evaluate the role of TLR2 in age-related inflammatory response to sepsis, we analyzed inflammatory cytokines in the myocardium and plasma of WT and TLR2 KO mice of different age. Levels of TNF-α, IL-1β, IL-6 and MCP-1 in the myocardium and plasma were markedly increased following CLP in WT mice, particularly one day following CLP, and old mice had much higher levels than young adult mice (Figure 4). At this time point, IL-6 levels in the myocardium and plasma of old mice were approximately 3 folds of those in young adult mice. The levels of TNF-α, IL-1β and MCP-1 in the myocardium and plasma also had significant age differences (Figure 4). The levels of inflammatory cytokines subsequently declined in both young adult and old WT mice. Four days after CLP, the levels of inflammatory cytokines in the myocardium and plasma of young adult WT mice were close to the sham control levels. However, the levels in old WT mice remained significantly higher than those in their sham controls (Figure 4). Apparently, aging-related hyper-inflammatory response is correlated with worse cardiac dysfunction and high mortality in old WT mice with sepsis.

TLR2 KO reduced inflammatory cytokine levels in the myocardium and plasma of both young adult and old mice with sepsis (Figure 4). However, the decline was more pronounced in old animals. For example, old TLR2 KO mice displayed a greater than 50% reduction in myocardial and plasma levels of IL-6 from the levels in old WT mice at 1 day after CLP. In contrast, young adult TLR2 KO mice had a 25%–27% reduction in IL-6 levels in the myocardium and plasma from the levels in young adult WT mice at 1 day after CLP (Figure 4). The levels of TNF-α, IL-1β and MCP-1 in the myocardium and plasma also exhibited greater reduction in old TLR2 KO mice when compared to young adult TLR2 KO mice. It is noteworthy that TLR2 KO markedly diminished the difference in the inflammatory activity between old and young adult septic mice. Thus, TLR2 mediates the inflammatory response to sepsis and occupies a major role in elevating the inflammatory activity in old septic mice.

### TLR2 KO attenuates cardiac dysfunction and reduces mortality in old septic mice

There was no notable variance in heart function observed between old WT mice and old TLR2 KO mice subjected to sham treatment. Cardiac function was markedly improved in old TLR2 KO mice following CLP (Table 2). In comparison to old WT mice, old TLR2 KO mice had better developed pressure (62 ± 6 vs. 50 ± 5 mmHg; *P *< 0.05), ejection fraction (26 ± 3 vs. 18 ± 2.0%; *P *< 0.05) and cardiac output (3.1 ± 0.3 vs. 2.4 ± 0.3 ml/min; *P *< 0.05) one day after CLP. At 2 and 4 days after CLP, old TLR2 KO mice displayed greater recovery of LV function than old WT mice. The cardiac function parameters in old TLR2 KO mice had recovered to levels slightly lower than baseline 4 days after CLP (Table 2).

**Table 2:** TLR2 KO improves cardiac function following CLP in old mice.

	WT sham	WT CLP 1 day	WT CLP 2 days	WT CLP 4 days	TLR2 KO sham	TLR2 KO CLP 1 day	TLR2 KO CLP 2 days	TLR2 KO CLP 4 days
Heart rates (bpm)	435 ± 41	420 ± 42	410 ± 52	430 ± 21	446 ± 41	454 ± 38	445 ± 51	450 ± 43
Developed pressure (mmHg)	82.4 ± 5.1	50.5 ± 5.2^a^^b^	54.3 ± 4.3^a^^b^	67.4 ± 4.4^a^^b^	82.5 ± 7.4	62.5 ± 6.5^a^^b^	65.4 ± 5.8^a^^b^	70.2 ± 5.5
End-systolic volume (ul)	8.1 ± 0.7	27.3 ± 3.2^a^^b^	24.0 ± 2.1^a^^b^	16.0 ± 1.3^a^^b^	8.3 ± 0.7	19.5 ± 2.2^a^^b^	15.5 ± 1.3^a^^b^	12.0 ± 2.0^a^^b^
End-diastolic volume (ul)	20.0 ± 1.7	33.4 ± 3.1^a^	30.1 ± 2.5^a^	24.0 ± 2.0^a^	21.4 ± 1.6	25.5 ± 3.1^b^	22.5 ± 3.0^b^	22.0 ± 2.1
Ejection fraction (%)	61.7 ± 5.3	18.2 ± 2.1^a^^b^	20.6 ± 3.2^a^^b^	33.4 ± 2.3^a^^b^	62.1 ± 5.2	26.0 ± 3.1^a^^b^	31.5 ± 3.2^a^^b^	45.7 ± 4.2^a^^b^
Cardiac output (ml/min)	5.4 ± 0.5	2.4 ± 0.3^a^^b^	2.5 ± 0.1^a^^b^	3.4 ± 0.2^a^^b^	5.5 ± 0.7	3.1 ± 0.3^a^^b^	3.2 ± 0.2^a^^b^	4.5 ± 0.2^a^^b^

Old WT and TLR2 KO mice (18–20 month old) were subjected to CLP or sham procedure. Cardiac function was analyzed 1–4 days following CLP. Data are expressed as mean ± SE.

*n* = 6 in each group.

^a^ *P *< 0.05 vs. respective sham.

^b^ *P *< 0.05 vs. WT old at the same time point.

**Figure 5:**
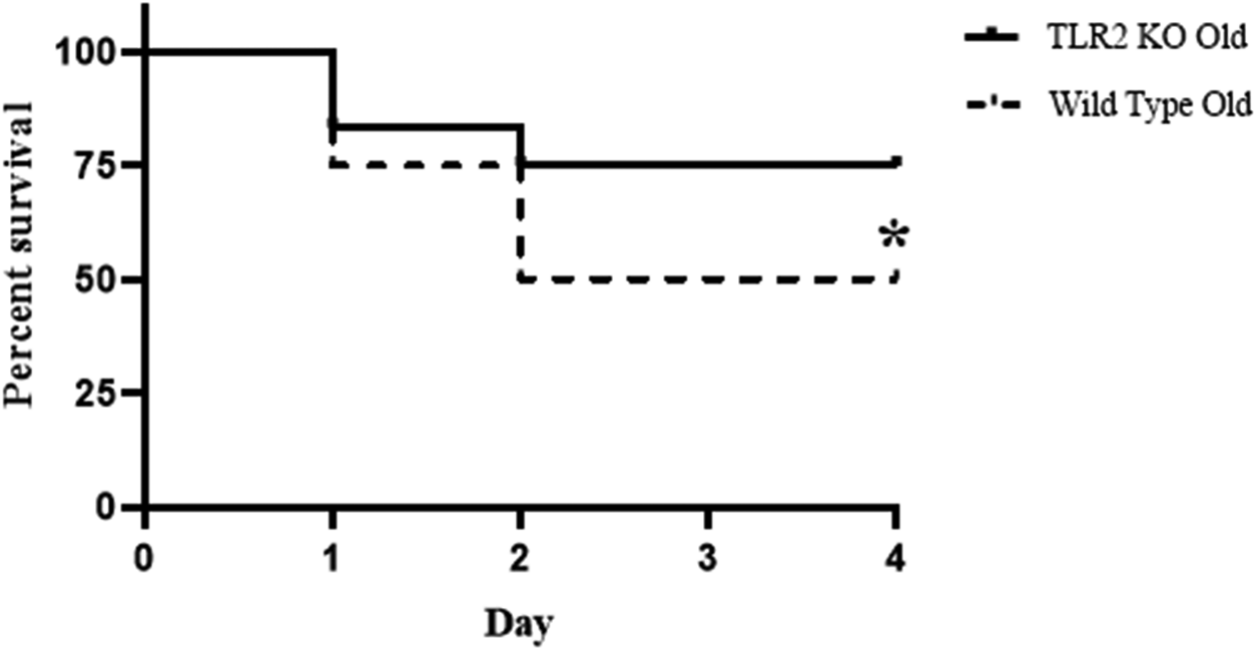
TLR2 KO decreases mortality rate during sepsis in old mice. WT old mice and TLR2 KO old mice were subjected to CLP. Mortality was reduced to 25% during the 4-day period in TLR2 KO old mice compared to WT old mice with a 50% mortality. *P < 0.05 *vs.* WT old, *n* = 12 in each group.

Previous study found that anti-TLR2 treatment improved survival in young mice with severe sepsis (15). In the present study, we found that TLR2 KO improved cardiac function in old septic mice, resulting in an improved survival rate. Compared to the 50% survival rate in old WT mice after CLP, the survival rate in old TLR2 KO mice was improved to 75% (Figure 5). Thus, TLR2-mediated myocardial hyper-inflammation is responsible for the worse cardiac dysfunction and higher mortality in old septic mice.

### Old mice display greater myocardial inflammatory activity and cardiac dysfunction following TLR2 stimulation

**Figure 6:**
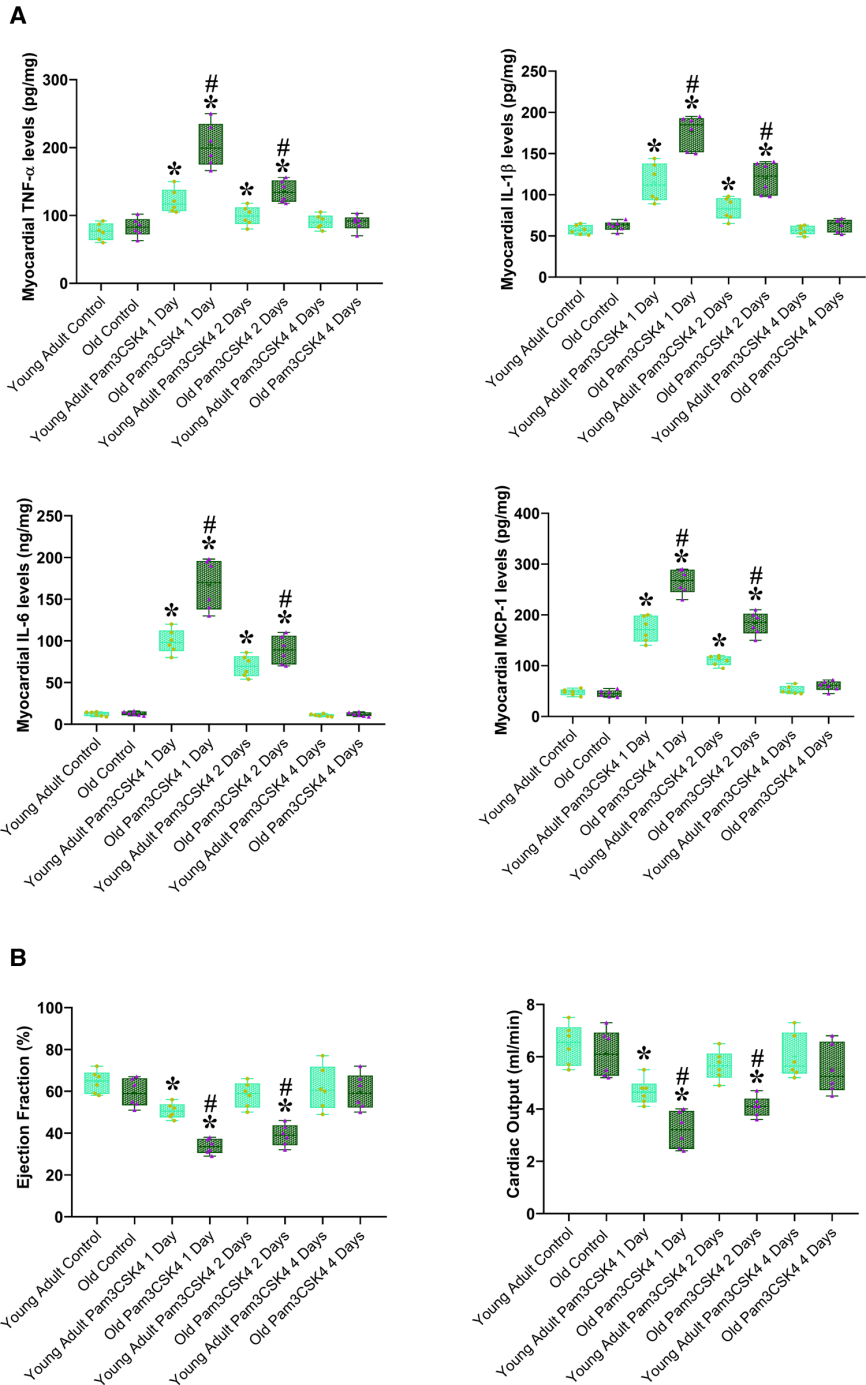
Aging exaggerates the inflammatory activity and worsens cardiac dysfunction caused by TLR2 stimulation. Young adult and old WT mice were treated with the specific TLR2 agonist Pam3CSK4 (2.5 µg/ g, iv) or normal saline. TNF-α, IL-1β, IL-6 and MCP-1 in the myocardium were analyzed using ELISA, and cardiac function was assessed using microcatheter 1, 2 and 4 days after treatment. (**A**). Myocardial levels of inflammatory cytokines increased in both young adult and old mice at 1 and 2 days after administration of TLR2 agonist, and old mice displayed a greater increase in comparison to young adult mice. (**B**) Ejection fraction and cardiac output decreased in young adult and old mice at 1 and 2 days after administration of TLR2 agonist in comparison to control levels, and old mice had worse cardiac function in comparison to young adult mice. Data are presented as box-and-whiskers plots where the upper and lower borders of the box represent the upper and lower quartiles; the horizontal line inside the box represents the median; the upper and lower whiskers represent the maximum and minimum values of nonoutliers; and the + sign represents the mean, *P < 0.05 vs. corresponding control, ^#^*P *< 0.05 vs. young adult at the same time point. *n* = 6 in each group.

To determine the role of elevated myocardial TLR2 level in aging-related exacerbation of myocardial inflammatory activity and cardiac dysfunction in sepsis, we determined myocardial inflammatory response to TLR2 agonist and associated cardiac functional change in young adult and old WT mice. After administration of the specific TLR2 agonist Pam3CSK4 (2.5 µg/ g, iv), we examined LV function and myocardial levels of inflammatory cytokines at 24, 48 and 96 h. Figure 6A shows that myocardial levels of TNF-α, IL-1β, IL-6 and MCP-1 in both young adult and old mice increased at 24 and 48 h in response to the TLR2 agonist. Notably, old mice had much higher levels of these inflammatory cytokines in the myocardium at 24 and 48 h in comparison with young adult mice. Thus, increased levels of TLR2 in the myocardium of old mice enhance myocardial inflammatory activity following TLR2 stimulation.

Cardiac dysfunction correlated to myocardial production of cardiodepressant cytokines. Ejection fraction and cardiac output decreased at 24 and 48 h after the exposure to Pam3CSK4, and old mice had worse cardiac function (Figure 6B). Interestingly, cardiac function was recovered after 96 h in both young adult and old mice as myocardial cytokine levels was normalized. It appears that aging enhances myocardial inflammatory response to TLR2 stimulation, and elevated concentrations of cardiodepressant cytokines in the myocardium resulted in greater cardiac function deficit in old mice.

## Discussion

Sepsis is defined as a systemic inflammatory response syndrome triggered by an infection (16). The incidence of sepsis is greater in the elderly population, and elderly patients with sepsis often have more severe organ dysfunction and higher mortality rate (3). While the higher mortality in older septic patients is closely associated with organ dysfunction, the underlying mechanism responsible for aging-related susceptibility to organ dysfunction remains incompletely understood. The present study found that CLP-induced sepsis caused greater cardiac dysfunction in old mice in comparison to young adult mice, resulting in a high mortality. Noticeably, worse cardiac dysfunction and mortality in old septic mice were associated with excessive inflammatory activity correlated to elevated TLR2 levels. Knockout of TLR2 attenuated myocardial and systemic inflammatory activity, significantly improved cardiac performance and decreased mortality in old septic mice. Thus, elevated TLR2 level/activity mediates inflamm-aging, resulting in exaggerated inflammation and cardiac dysfunction during sepsis in old mice. TLR2 appears to be a therapeutic target for suppressing inflammatory activity and protecting cardiac function in old subject in sepsis.

Elderly patients with sepsis frequently develop multiple organ failure that accounts for a poor prognosis (17). While heart failure is a significant risk factor for mortality in sepsis (18), the impact of aging on septic cardiac dysfunction is not well defined. We observed in the present study that moderate sepsis induced by CLP causes cardiac dysfunction in both young adult mice and old mice. However, old mice exhibit more severe cardiac dysfunction with much lower LV ejection fraction and cardiac output when being subjected to such moderate sepsis. The impact of aging on cardiac dysfunction observed in this sepsis model is similar to that observed in endotoxemia models (10, 19). As a result, 50% of old septic mice die while all young adult septic mice survive following an identical CLP protocol. It is noteworthy that old mice die in the first 2 days of sepsis when their LV ejection fraction is declined to 20% or lower, and no death occurs after 2 days when cardiac function is improving. Apparently, severe cardiac dysfunction and related ischemic injury/dysfunction in other organs are the major cause of death in old septic mice.

Our previous study found that the myocardium of old mice has higher level of TLR2 (13), and the present study confirmed this observation and identified elevated TLR2 levels in the liver and kidney. Based on these findings, we tested the hypothesis that elevated TLR2 levels augment inflammatory activity in sepsis and thereby results in worse cardiac dysfunction. Clearly, old septic mice have greater levels of inflammatory cytokines, including TNF-α, IL-1β, IL-6 and MCP-1, in the myocardium and plasma at all time points examined. These inflammatory cytokines, particularly TNF-α, IL-1β and IL-6, are well known cardiodepressant factors (9, 11), and TNF-α and IL-1β synergistically depress myocardial contractility (20, 21). Greater MCP-1 level is also correlated with worse cardiac dysfunction. Our previous study demonstrates that MCP-1 promotes monocyte infiltration to the myocardium in old endotoxemic mice and thereby mediates worse cardiac dysfunction (10). In addition, lowering MCP-1 level could decrease the levels of other inflammatory mediators and improve survival rate in sepsis (22, 23). Thus, all of the four inflammatory cytokines play a mechanistic role in cardiac dysfunction caused by sepsis. Augmented production of these cardiodepressant factors is responsible for the worse cardiac performance in the old septic mice.

In clinical settings, efforts to treat sepsis and septic shock by targeting the acute inflammatory response with anti-inflammatory agents have not resulted in improved patient outcomes (24). It is crucial to note that most patients in these clinical trials were gerontal patients. The immune system of the elderly differs from that of younger adult patients, including alterations in the innate immunity (25). Therefore, understanding of the mechanism of aging-related pathobiology of sepsis will lead to the development of effective strategies to inhibit inflammatory response in elderly subjects.

The results of the present study show that TLR2 KO has a greater effect on the production of inflammatory mediators in old mice. While both young adult and old TLR2 KO mice have reduced levels of TNF-α, IL-1β, IL-6 and MCP-1 in the myocardium and plasma after CLP compared to age-matched WT mice, old TLR2 KO mice exhibited a much greater reduction in inflammatory mediators compared to young adult TLR2 KO mice. Thus, TLR2 plays an important role in exacerbation of the inflammatory activity in sepsis. Accompanying the significant reduction of cytokine production, LV performance is markedly improved in old TLR2 KO mice at 24 h after CLP compared to old WT mice. Cardiac output is 30% greater in old TLR2 KO mice in comparison to old WT mice. The improved cardiac function in old TLR2 KO mice resulted in higher survival rate (75% vs. 50% in old WT mice) during the 4-day period after CLP. These findings not only demonstrate the role of TLR2 in mediating the inflammatory activity and cardiac dysfunction in old septic mice, but also support the notion that cardiac dysfunction is a major risk factor for death. These findings provide preclinical evidence that TLR2 blockers is beneficial for sepsis patients, especially in the elderly.

It should be noted that LV performance is not recovered to the baseline level 4 days after CLP in old TLR2 KO mice. This suggests that TLR2-independent mechanism(s) also contribute to aging-related cardiac dysfunction in septic mice. In this regard, we observed in a previous study that old mice exhibit myocardial hyper-inflammatory response and exacerbated cardiac dysfunction when being exposed to endotoxin (Li X, Aging in press). It is likely that inflamm-aging involves the alteration of other TLRs, including TLR4. A recent report shows that older rats (7 month old) have greater oxidative stress in multiple organs, including the heart, following CLP (26). Thus, inhibition of TLR2 alone would attenuate, rather than abrogate, cardiac dysfunction in old septic subject.

TLR2 is a component of the innate immune system and plays a crucial role in mediating inflammatory response to polymicrobial sepsis. Released PAMPs and DAMPs during sepsis bind to the TLR2 receptor on monocytes, macrophages and other pro-inflammatory cells, initiating an intracellular signaling pathway leading to NF-κB activation, assembly of the inflammasome, and the subsequent generation of inflammatory cytokines (27). However, excessive activation of theTLR2 signaling pathway contributes to tissue damage, organ failure, and even mortality during sepsis. Previous studies have shown that aging is an independent predictor of mortality in sepsis (4, 28), and elevated TLR2 activity in the aging heart exacerbates myocardial inflammatory activity (13). In the current study, we observed that TLR2 activity was greater in the hearts of old WT mice compared with young WT mice when using a specific TLR2 agonist, PAM3CSK4, to stimulate this innate immune receptor. Both young and old mice showed increased levels of TNF-α, IL-1β, IL-6 and MCP-1 in the myocardium at 24 and 48 h after the treatment. However, old WT mice exhibited greater inflammatory activity. As a result, old WT mice had worse ejection fraction and cardiac output at these time points. Interestingly, myocardial levels of inflammatory mediators and LV function parameters returned to the baseline levels 4 days after the exposure to PAM3CSK4. This indicates that a single dose of TLR2 agonist elicits an inflammatory response lasting for a shorter time while sepsis induces a longer-lasting inflammatory response through sustained TLR2 stimulation by bacterial products. Nevertheless, elevated TLR2 levels in old mice play a major role in exacerbation of inflammatory activity and cardiac dysfunction following TLR2 stimulation. Together, the results demonstrate that inflamm-aging involves elevation of TLR2 level in vital organs and higher TLR2 levels drives greater inflammatory activity. The findings support the concept that elevated TLR2 level exacerbates the inflammatory activity to drive the severity of cardiac dysfunction in old subjects with sepsis.

It is noteworthy that myocardial cytokine levels after CLP in TLR2 KO old mice are higher than sham controls and Pam3CSK4-treated old mice. This observation indicates that TLR2-independent mechanism is involved in inducing and/or maintaining myocardial inflammation in sepsis. In this regard, polymicrobial sepsis also induces the activation of TLR4, TLR9 and RAGE (29–32). These pattern-recognition receptors could play a role in elevation of cytokine levels. Future studies are needed to identify TLR2-independent mechanism that contributes to aging-related exacerbation of inflammation in sepsis.

It should be noted that the present study has focused on the impact of aging on the host with an emphasis on innate immunity. Two recent studies (33, 34) have reported that aging alters the stability and/or potency of gut bacteria, and such an effect of aging might modulate the severity of sepsis caused by bacteria of gut origin. Further studies are warranted to identify the factors from gut bacteria of old subjects that exacerbate sepsis severity.

## Conclusion

Elevated levels of TLR2 in multiple organs of old mice augment the expression of cardiodepressant cytokines TNF-α, IL-1β, IL-6 and MCP-1 during sepsis, leading to greater cardiac dysfunction and high mortality. TLR2 KO markedly attenuates the inflammatory activity in old septic mice, diminishing the age-related difference in inflammatory activity. This effect of TLR2 KO results in improved cardiac function at all time points during sepsis in old mice and reduces their mortality. In addition, old WT mice exhibit greater myocardial inflammatory activity and cardiac dysfunction following an exposure to TLR2 agonist. Thus, elevated levels of TLR2 in multiple organs, including the heart, drive sepsis severity in old mice. Modulation of the level or activity of TLR2 in old septic subjects may improve their outcome.

## Data Availability

The raw data supporting the conclusions of this article will be made available by the authors, without undue reservation.
